# Transparent Electromagnetic Shielding Film Utilizing Imprinting-Based Micro Patterning Technology

**DOI:** 10.3390/polym13050738

**Published:** 2021-02-27

**Authors:** Hyun-Seok Choi, Su-Jeong Suh, Sang-Woo Kim, Hyun-Joong Kim, Ji-Won Park

**Affiliations:** 1 Advanced Materials Science and Engineering, SungKyunKwan University, Suwon-si 16419, Korea; hschoi@smtcore.com (H.-S.C.); suhsj@skku.edu (S.-J.S.); 2 Clean Energy Research Center, Korea Institute of Science and Technology, Seoul 02792, Korea; swkim@kist.re.kr; 3 Research Institute of Agriculture and Life Sciences, College of Agriculture and Life Sciences, Seoul National University, Seoul 08826, Korea; 4 R&D Center of JB Lab Corporation, Seoul 08826, Korea

**Keywords:** electromagnetic interference shielding, transparency, ultraviolet imprinting, soft molding, flexible structure

## Abstract

Utilization of methods involving component integration has accelerated, owing to the growth of the smart mobile industry. However, this integration leads to interference issues between the components, thereby elucidating the importance of the electromagnetic interference (EMI) shielding technology to solve such issues. EMI shielding technology has been previously implemented via the reflection or absorption of electromagnetic waves by using conductive materials. Nevertheless, to tackle the recent changes in the industry, a transparent and flexible EMI shielding technology is necessitated. In this study, a transparent and flexible EMI shielding material was fabricated by filling a conductive binder in a film comprising an intaglio pattern; this was achieved by using the ultraviolet (UV) imprinting technology to realize mass production. Subsequently, changes in the aperture ratio and shielding characteristics were analyzed according to the structure of the pattern. Based on this analysis, a square pattern was designed and a film with an intaglio pattern was developed through a UV imprinting process. Furthermore, it was confirmed that the transmittance, conductivity, and EMI shielding rate of the film were altered while changing the coating thickness of the conductive particles in the intaglio pattern. The final film prepared in this study exhibited characteristics that satisfied the required EMI shielding performance for electric and electronic applications, while achieving flexible structural stability and transparency.

## 1. Introduction

Smart mobile devices are on the verge of becoming the centers of modern industry and culture due to the fact that various functions and services are being implemented in a single system, while characteristics of various systems are integrated into one device. With the functional development of smart mobile products, multifunctionalities and integration of different parts and materials are indispensable [1,2].

However, the integration of diverse materials and parts is also known to cause various problems. Even if the systems are integrated, each system operates using its independent processes. Therefore, system interference caused by electromagnetic waves during system operation leads to difficulties associated with product manufacturing [3,4]. This is a problem that must be solved because it can not only lead to a malfunction of the entire product, but can also induce electromechanical defects such as overcurrent. In particular, distortion of the display, radio interference, and noise generation are typical problems associated with electromagnetic interference, which act as critical issues with regard to the applicability of the final product [5,6].

In particular, the integration of technologies required by the convergence industry has the following technical characteristics, making it more difficult to solve problems such as electromagnetic interference [1,7,8].

1. Complex functionality required while miniaturization;
2. Flexible or elongated design rather than a fixed system;
3. Various interfacial issues arise due to diversification of materials.

Electromagnetic waves are waveforms that perpendicularly intersect the electric and magnetic fields and are essentially generated by the flow of electric current. In order to control the interference provided by electromagnetic waves, various electromagnetic shielding technologies have been recently studied. Electromagnetic wave shielding implies that a certain part of the device space is surrounded by a conductor or a ferromagnetic material to ensure that either the interior is not affected by the external electromagnetic field, or that the electromagnetic field generated inside does not project outwards [9,10,11]; the higher the frequency of the electromagnetic field, the more effective it is. Therefore, the degree of shielding of electromagnetic waves depends on the material used and its thickness, size of the space to be shielded, and frequency of electromagnetic waves to be blocked. The attenuation rate of electromagnetic waves has been described in the following equation, according to the electromagnetic wave shielding technology:

(1) $$\mathrm{EMI}\;\mathrm{Shielding}\;\mathrm{Effectiveness},\;\mathrm{SE}\left(\mathrm{dB}\right)=SE_{a}+SE_{r}+SE_{m}=10\log\left[\frac{P_{i}}{P_{0}}\right]=20\log\left[\frac{E_{i}}{E_{0}}\right]$$

where SE_a_ is the absorption shielding effectiveness, SE_r_ is reflection shielding effectiveness, and SE_m_ is multiple internal reflections. E_i_ is the electric field strength at the initial stage and E_o_ is the electric field strength after the incorporation of shield materials. The electromagnetic wave attenuation rate is expressed in dB and is calculated as the sum of the absorbance and reflectance of the electromagnetic wave. A difference of 10 dB implies a 90% attenuation of the electromagnetic waves at the interior and exterior, and a 99% attenuation in the case of 20 dB. In general, when a barrier film of a metal material is formed, it exhibits a very strong attenuation rate; further, it is known that a copper sheet covering exhibits a shielding performance that exceeds 100 dB. In general, the shielding performance for industrial applications is known to be approximately 40–50 dB [3,12,13].

With an increase in the use of smart devices and further development of their functions, the transparency and flexibility of packaging materials is considered important [14,15]. According to this trend in the industry, the use of flexible and transparent shielding materials is also necessitated. Various systems for coating conductive polymers and metal particles have been employed to realize transparent and flexible conductors [16,17]. When a conductive polymer is used, transparency and flexibility are achievable, but durability is problematic. Recently, various studies have been carried out to form a conductive pattern using silver and copper nanowires [18,19,20]; however, the conductivity and shielding properties tend to be limited. To solve this problem, a hybrid system that utilizes two r more technologies has been proposed. In order to control transparency, patterning research and techniques for complex implementation of not only shielding performance but also heat dissipation performance are being introduced [3,21,22]. Furthermore, research on a coating system using conductive particles has been carried out; it is revealed that changes in conductivity tend to occur according to the binder and curing properties. In order to maximize shielding performance, it is important to maximize the conductivity of the conductive composite [23]. In addition, in general, the proportion of conductive particles increases, but the flexibility decreases. Therefore, the structure is destroyed when it is forcibly deformed, and a space design is required to protect such a fractured structure. In order to design such a space, a technique for forming intaglio patterns is being introduced [24,25].

This study focused on filming technology among existing shielding technologies. In particular, we designed a process that can realize transparent/shielding/flexible characteristics at the same time and looked at the reproduction process of the technology throughout the entire process. The step of forming a pattern to implement soft molding and the search for the optimal pattern structure through simulation, evaluation of resin to confirm the UV curing system, evaluation of conductive properties of conductive particles, and fabrication using patterned film through soft molding we would like to explain the overall research on the design and mass production process of the system, up to the basic performance evaluation of an EMI shielding film.

## 2. Experimental

### 2.1. Concept of Transparent/Flexible Electromagnetic Wave Shielding Film Manufacturing

**Figure 1:**
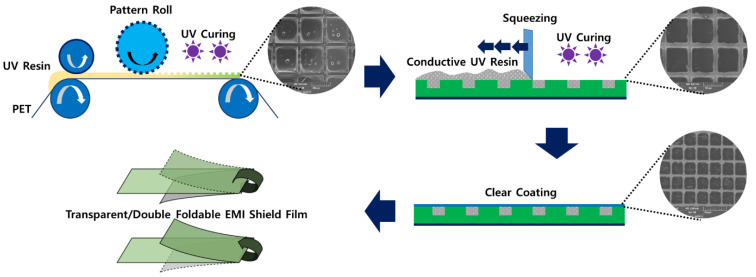
Schematic of the entire manufacturing process of a transparent and flexible electromagnetic interference (EMI) shielding film.

Micropatterning technology is that repeatedly implements a finely repetitive pattern on a surface. In the case of systems that do not consider repetitive production, it is common to form a precise pattern structure through stamping, but this technique cannot be applied to the mass production process. In addition, since the finally required system must have a flexible structure, a polymer film must be used as a support. It is a technology that is very difficult and expensive to form a precise pattern on the surface of such a polymer film. Soft molding technology applied with UV imprinting process was used to form a flexible pattern structure while enabling continuous production. By applying soft molding technology, large area continuous patterning can be formed at high speed. The manufacturing process of transparent conductive film using micropatterning and imprinting technology is shown in Figure 1. The micropatterning process using imprinting consists of the following sequence.

A pattern of a desired shape is formed on a metal panel that is advantageous to form a pattern through etching. In order to transfer the formed pattern to a film, etc., a working pattern is remanufactured. The final pattern is not manufactured from the thus manufactured working pattern. An important point in the soft molding technique is to manufacture a pattern film once again to form a final pattern using a working pattern, and to form a final UV pattern using the film.

Since this film is applied to an intermediate process, a large area pattern can be formed, and contamination of the working pattern can be minimized. Additionally, since the film is fixed until the pattern is completely formed, the uniformity and stability of the pattern are improved. In this system, a binder is coated on the entire surface of the engraved film. Additionally all the binders were removed through a squeeze method, except for the portion with the intaglio pattern wherein only a composite-type binder can be coated. Thereafter, photo-curing and heat-curing were employed to prepare the final film.

### 2.2. Preparation of the UV Pattern Layer

**Figure 2:**
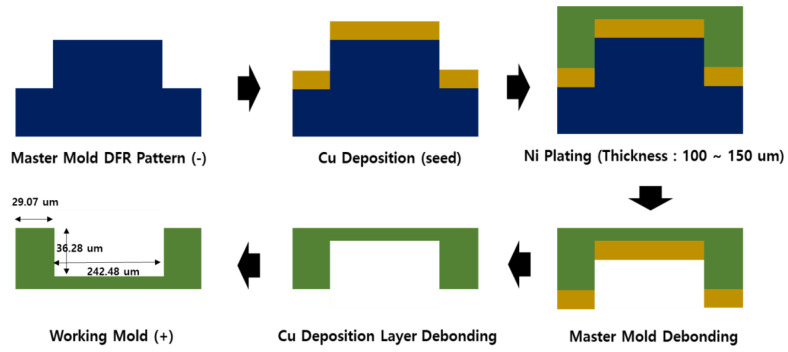
Manufacturing process of working pattern mold for imprinting. **Blue**—master pattern, **Yellow**—copper coating, **Green**—nickel plating.

“Soft Molding” technology is a large area pattern technology using film. This is a technology that copies the design of a master pattern made of metal, etc., into a film and coats a curable resin on the pattern to create the final material on which the pattern is formed. In order to realize the soft molding technology, the manufacturing of a master pattern and an elaborate transfer process of the pattern are required (Figure 2).

A master mold was fabricated using a dry film resist (DFR) to produce an intaglio pattern comprising partition walls. The working mold was manufactured by using Cu as a seed layer and the master mold. Nickel plating was performed on the master mold with Cu as the base layer. The thickness of the nickel stamper was altered to 250 μm, and its back was polished to be flat.

The previously manufactured working mold exhibited an embossed pattern with the partition wall thickness of 36.27 μm, partition wall height of 29.07 μm, and opening size of 242.47 μm. There was a deviation of approximately ±10, 4, and 5 μm in the thickness, height, and opening size of the mold manufactured using the DRF pattern, respectively.

For UV imprinting, PET (SKC, SH86, thickness 100 μm) coated with a urethane primer was used as the base film because it adhered to the UV resin. This resin was prepared by blending a photocurable monomer, a photocurable oligomer, a photoinitiator, and an additive into a paste and subsequently stirred at a high speed (1500 rpm) for 3 min. The lamination was carried out by applying the UV resin using an embossed soft mold and subsequently compressing it using a rubber roll; next, dose curing was carried out at the rate of 2000 mJ/cm^2^ under low- and medium-pressure UV lamps and peeling was performed. 2-ethylhexyl acrylate (Sigma Aldrich, St. Louis, MO, USA) and acrylic acid (Sigma Aldrich, St. Louis, MO, USA) were used as photocurable monomers; epoxy acrylate (PE210, Miwon Specialty Chemical, Seoul, Korea) and urethane acrylate (PU640, Miwon Specialty Chemical, Korea) were used as photocurable oligomers. 2-hydroxy-2-methyl propiophenone (Irgacure 1173, BASF, Ludwigshafen, Germany) was used as the photoinitiator. The ratio of each material is composed of 2-EHA:AA:PE210:PU640:Irgacure 1173 = 57:3:20:20:1.

For patterning using a soft mold, embossing can be carried out via imprinting the working mold (+) twice (Table 1). This can be costly and time-consuming as compared to manufacturing a replica mold using a simple imprinting process, wherein there is no need to additionally manufacture an expensive Ni working mold.

Patterns are a preprocess for creating spaces and do not create shielding properties by themselves. The EMI shielding effect is expressed by filling a conductive material in the void area made through soft molding.

### 2.3. Electrical Conductivity

Conductivity was measured with the aim of evaluating the surface resistance. Furthermore, it is well known that the sheet resistance is inversely proportional to the shielding performance [26,27]. Therefore, in order to measure the sheet resistance of the neat type conductor, a surface resistance meter (SCC-625; ASTM-257, 3M, Saint Paul, MN, USA) was used. The surface resistance of the patterned film was measured via the 4-probe method (MCP-T610, Mitsubishi chemical, Tokyo, Japan). The shielding performance of the film was evaluated using a network analyzer (E8364D, Agilent, Santa Clara, CA, USA). The experiment was conducted under 1 GHz condition. Further, S-parameter measurements were performed according to the ASTM D4935 (KS C 0304) standard. In the material review stage, the change in sheet resistance was directly measured using the ASTM-257 technique. After the pattern was formed, the change in resistance was measured through the 4-probe method, and the change in shielding characteristics was observed through it.

### 2.4. Aperture Rate and Shield Effectiveness

The aperture ratio was calculated by assuming that the conductor region completely blocked the incoming light during the design process. The shield effectiveness was calculated through simulation using the ANSYS HFSS simulator that employs the finite element analysis (FEM) technique. The simulation of the single-structure shielding film was modeled by using silver as a mesh structure at a conductivity of 2 × 10^6^ S/m.

### 2.5. Binder Curing Rate

Photodifferential scanning calorimetry (Photo-DSC) was used to examine the curing rate. The instrument (Q-200; TA instrument, New Castle, DE, USA) employed a metal light source at the condition of 10 mW/cm^2^, and measured the curing rate at room temperature.

### 2.6. Flexure Test

Among the various applications of transparent shielding films, their utility in wearable/flexible devices has been the most significant. Flexibility is one of the less important performance parameters of existing smartphones and displays; however, it is recognized as the most important factor when manufacturing wearable devices [28,29]. Therefore, the adhesion reliability between the intaglio pattern of the transparent shielding film and the conductive layer was evaluated. In particular, the technique used for evaluating flexibility is also important; in this study, this evaluation was performed by employing equipment that can bend up to an angle of 135°, and the bending angle can differ by varying the bending radius from 1R to 5R. Herein, the bending angle was determined to be 60°, and changes in the surface resistance were confirmed after performing 150 evaluations.

## 3. Results and Discussion

### 3.1. Design of the Transparent Shielding Film

**Figure 3:**
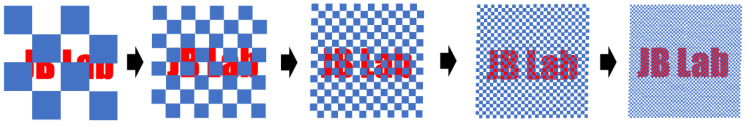
Difference in transmittance according to the change in pattern size change (total aperture ratio is the same as 50%).

There are two methods to increase transparency when incorporating fillers into the substrate. The most commonly used method is to reduce the size of the filler particles to such a level that they do not interfere with visible light. This approach has been used in various fields by employing nanosized particles. However, there are numerous difficulties associated with dispersion of particles, including the reduction in conductivity that occurs because the bonds between particles are broken due to dispersion. The second approach is to form a fine pattern. Even if they have the same aperture ratio as shown in Figure 3, the perceived image changes with the pattern size, and we generally perceive that transparency has been achieved. In addition, when the precision of the pattern is increased, the pattern can be fabricated with a more delicate width to ensure that the visible light transmittance can be increased.

**Figure 4:**
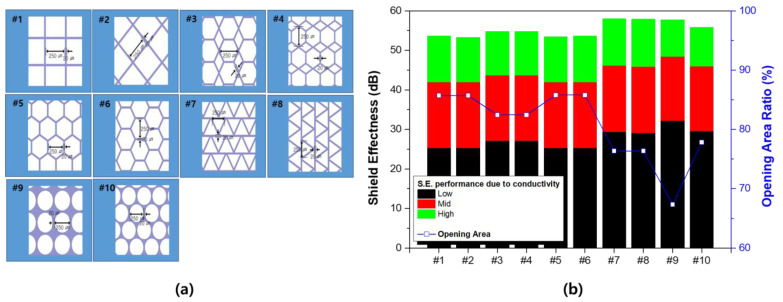
Evaluation of the aperture ratio and shield effectiveness according to pattern design. (**a**) Pattern idea derived from the standard of 20 μm line width and 250 μm diameter of the opening area. (**b**) Light transmittance and shielding performance simulation result for each pattern (conductivity condition—low: 6.1 × 10^4^, mid: 6.1 × 10^5^, and high: 6.1 × 10^7^, Unit: S/m).

Various types of pattern designs were designed and the shielding characteristics and transmittance of each design were compared. This is to implement transparent/shielding characteristics at the same time and to review suitability in the actual mass production process. Since the process of making an actual pattern is very complex, we tried to examine each performance in advance through simulation and select a structure suitable for actual manufacturing. Figure 4 is the width of the open space was fixed at 250 μm and the line width of the pattern was fixed at 20 μm; subsequently, 10 different patterns were designed. The patterns exhibiting excellent electromagnetic wave shielding performance (#7 and #8) tended to show inferior optical properties. Samples with high aperture ratios exhibited no significant differences, even with regard to the actual shielding ratio (#1, #2, #5, and #6.).

**Figure 5:**
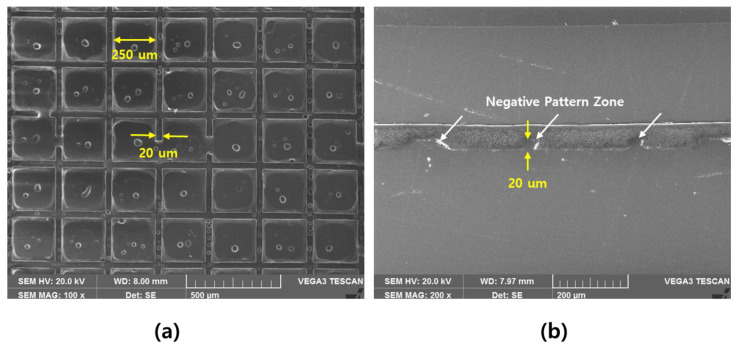
Enlarged image of the film patterned via imprinting. (**a**) Top view with ×100. (**b**) Sectional view with ×200.

Among the structures demonstrating the same aperture and shielding ratios, #1 and #2 were considered to represent the best pattern structure; this is because the smaller the pattern contact, the higher the structural stability of the pattern formation. Based on the simulation results, a pattern with a square structure was designed. The structure of the manufactured pattern is shown in Figure 5, and it can be confirmed that a region with a negative pattern was formed.

### 3.2. Design of the Electroconductive Binder System

A complex binder system was selected to form a conductive structure in the region where the negative pattern was formed. The composite binder system is capable of fixing the conductive particles via curing, after dispersing conductive particles in a curable resin to form a structure. Various particles can be utilized when a composite binder system is used, and since this system can be utilized in a state with flowability, conductivity can be imparted to the microcavity.

In order to maximize the shielding performance, conductivity must be increased. It is generally known that metals afford low resistances, particularly silver and copper (Table 2). The relative ease of controlling the shapes of silver particles renders them extremely suitable for the construction of composite materials. In this study, the conductivity and curing properties were verified by using three types of particles, namely: spherical-type particle (HAG-100S, Changsung, Korea), plate-type particle (FAG-100S, Changsung, Korea), and laminated-type particle (HAG-100H, Changsung, Korea).

**Figure 6:**
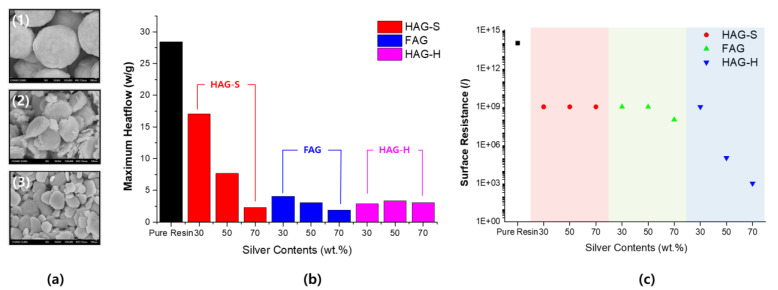
Curing properties and change in the surface resistance properties according to the type of metal particles. (**a**) SEM image of each silver particle applied to the conductive system 1:HAG-100S 2:HAG-100H 3:FAG-100S. (**b**) Reactivity in the UV curing process (peak of heat flow). (**c**) Comparison of surface resistance after UV curing.

Maximum heat flow is an indicator of the rate of reaction of each system during UV curing. Figure 6 shows the curing characteristics of each particle based on the absence of particles. It can be observed that the reaction rate decreased rapidly when particles are incorporated into the system. In the case of spherical particles, the reduction in the rate of reaction was small, even when the particles were mixed; however, in the case of plate-type and laminate-type particles, the reduction was significant. This phenomenon arose because the particles on the diffuse reflection sphere could penetrate the interior, owing to the diffuse reflection of light; however, the reflection at the surface was much more prevalent in the remaining two structures that formed a wide surface.

The experimental results of the surface resistances show an opposite trend to the results observed with the previously used curing rates. In the system using spherical particles, it was not possible to confirm the tendency of the resistance to decrease, even if the number of particles increased. In the system using plate-type silver particles, 30–50% of the results were the same as those of the system using spherical particles. On the other hand, when laminated-type particles were used, the results were completely different. In order to achieve suitable conductivity, an adequate contact between the particles is necessitated, and the probability of such contact is proportional to the ratio of the volume of the material. Therefore, a system with 30% material content is inadequate; further, resistance rapidly decreases for a system with 50% content. Only in the system with 70% material content, the resistance reached the threshold value. Since the spherical shape exhibits excellent spatial distribution, the contact probability is relatively low. On the other hand, a plate shape allows excellent contact probability; however, since the coating thicknesses are larger than the particle structures, the probability of the formation of a spatial lamination structure is high. Nevertheless, the shape of the laminate is widely considered advantageous for spatial contact due to significantly wider thicknesses at the left and right sides and a relatively increased overall thickness.

### 3.3. Micro Imprinting Film and Conductive Pattern

A conductive paste for filling the imprint pattern was prepared by using silver particles comprising a lamination structure, which showed the best surface resistance. Ag flakes were prepared by premixing an ester-based resin and an ether-based solvent using a powder with an average particle diameter (D50) of 0.76 μm. Subsequently, the filler was dispersed via a 3-roll milling method to prepare a paste. The Ag flake content was altered between 75 to 80 wt % to ensure a sheet resistance of 2 Ω/□.

**Figure 7:**
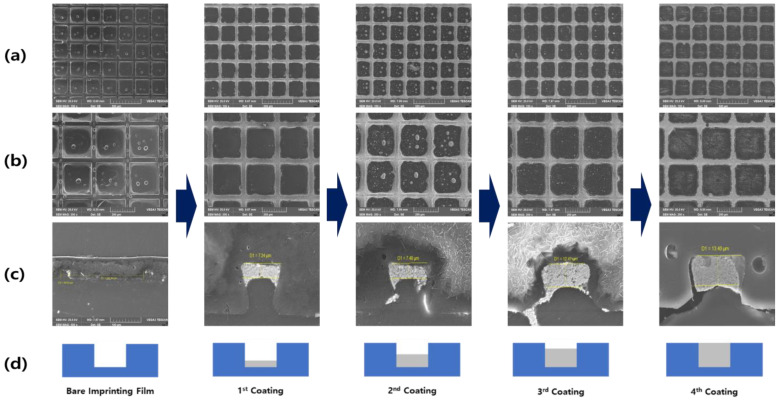
Change in shape according to pattern molding via coating of the conductive binder. (**a**) Top view with ×100. (**b**) Top view with ×200. (**c**) Sectional view ×3000. (**d**) Filling schematic diagram by coating step.

Figure 7 represents the scanning electron microscope image of a structure, in which a transparent film with am imprinted pattern and a conductive binder are layered in steps. A and B are the photographs taken from the top of the binder that is laminated on the structure with a negative pattern; C and D represent the measurement results of the cross section during the lamination process. When filling a structure that comprises a fine gap, a limited amount of filler can be added at a time. This structure exhibits a high viscosity and certain areas remained unfilled because of the presence of particles. Therefore, it was gradually filled through a continuous process. Coating processes were conducted four times in total, and the filling state and associated characteristics were examined in each step. It can be confirmed in each image that the filling amount of the conductive binder increases as the coating progresses.

**Figure 8:**
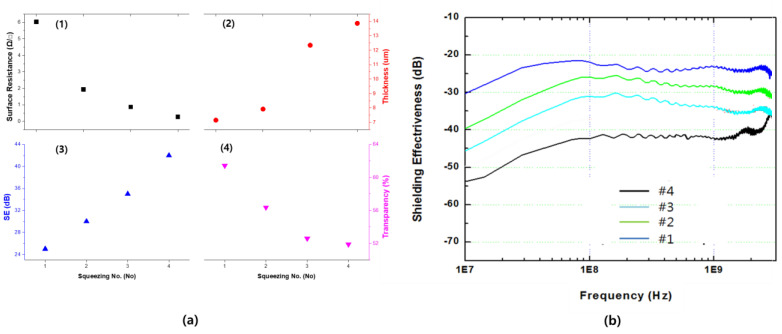
Evaluation of film properties and electromagnetic shielding properties according to coating conditions. (**a**) Main performance evaluation result by number of squeezing. 1: Surface resistance, 2: filling thickness, 3: shielding effectiveness at 1 GHz, and 4: transparency. (**b**) Shielding effectiveness change according to frequency change.

Figure 8 and Table 3 elucidate the filling thickness, surface resistance, electromagnetic shielding performance, and transparency, according to the filling stage of the binder. As the number of coatings increased, the thickness of the binder tended to increase gradually; the result of filling more than 90% of the imprinting space was observed in the 4th coating. As the thickness of the binder increased, the surface resistance decreased and the electromagnetic wave shielding performance tended to increase; further, the transparency decreased and tended to stabilize.

Thickness, conductivity, and shielding performance can be regarded as directly proportional to each other. The transmittance of the coated films tended to decrease significantly when compared to that of the bare film; however, it did not alter significantly with a change in the number of coatings. This result revealed the strength of the imprinting structure. Since the binder is only filled within the negative structure formed by imprinting, optical interference can be minimized. However, it appears that the damage to the surface and a decrease in transmittance are caused by the repeated coating processes and subsequent squeezing. Considering the light transmittance levels of the optical filters used in our daily lives, a transmittance range of 50–60% is considered extremely high.

## 4. Conclusions

In this study, an UV imprinting technology was used to manufacture an EMI shielding film that comprises a transparent/flexible structure. The intaglio pattern was designed in the opposite direction to that of the existing pattern, and it was possible to achieve an economical manufacturing process by applying the soft molding technique. It was confirmed that a difference in conductivity occurred depending on the structure of the particles, and an optimized particle was selected to form a pattern. Various designs were fabricated and different patterns were optimized for maximum performance and mass production. By adopting the intaglio structure, it was possible to stably fix the conductive particles; accordingly, excellent bending characteristics were obtained. Previous studies have indicated that the imprinting process involves expensive technologies; however, this study clearly exhibited that it is possible to develop an imprinting technology that is inexpensive and capable of mass production. Through research related to the entire process including material design, pattern design, introduction, and evaluation of mass production technology, it was possible to propose industrially applicable film processing technology. Therefore, it is expected that this novel process will be universally used to design parts for next-generation devices such as electronic wearables.

## Figures and Tables

**Table 1:** Evaluation of size change according to the molding process.

	Ni Working Mold	1st Soft Mold (−)	2nd Soft Mold (+)	3rd Soft Film (−)
Wall Thickness (μm)	36.276	30.549	30.549	30.411
Wall Width (μm)	29.069	29.954	26.772	20.593
Opening size	242.479	248.207	246.298	245.185

**Table 2:** Basic information of silver particles.

Test	HAG-100S	FAG-100S	HAG-100H
Tap Density (cc/g)	3.5	2.17	1.5–3
D50 (μm)	1.1	1.02	0.8
D100 (μm)	5.0	8.0	6.0
BET (m^2^/g)	0.85	2.4	2.8–3.2

**Table 3:** Evaluation of the changes in film properties according to coating conditions.

Test	Unit	1st Coating	2nd Coating	3rd Coating	4th Coating	Bare Film
Surface Resistance	Ω/□	6.028 × 10^0^	1.941 × 10^0^	8.731 × 10^−1^	2.813 × 10^−1^	
Binder Thickness	μm	7.15	7.92	12.34	13.86	
EMI Performance	dB	25	30	35	42	
Transparency	%	61.43	56.37	52.59	51.89	90.81

## Data Availability

The data presented in this study are available on request from the corresponding author.
